# Supplementary material for: Highly efficient and robust π-FISH rainbow for multiplexed in situ detection of diverse biomolecules
Source: Nat Commun. 2023 Jan 27;14:443. doi: 10.1038/s41467-023-36137-4 (PMC9883232; doi:10.1038/s41467-023-36137-4)
Supplement: Supplementary file 1 — Supplementary Information [file 41467_2023_36137_MOESM1_ESM.pdf]

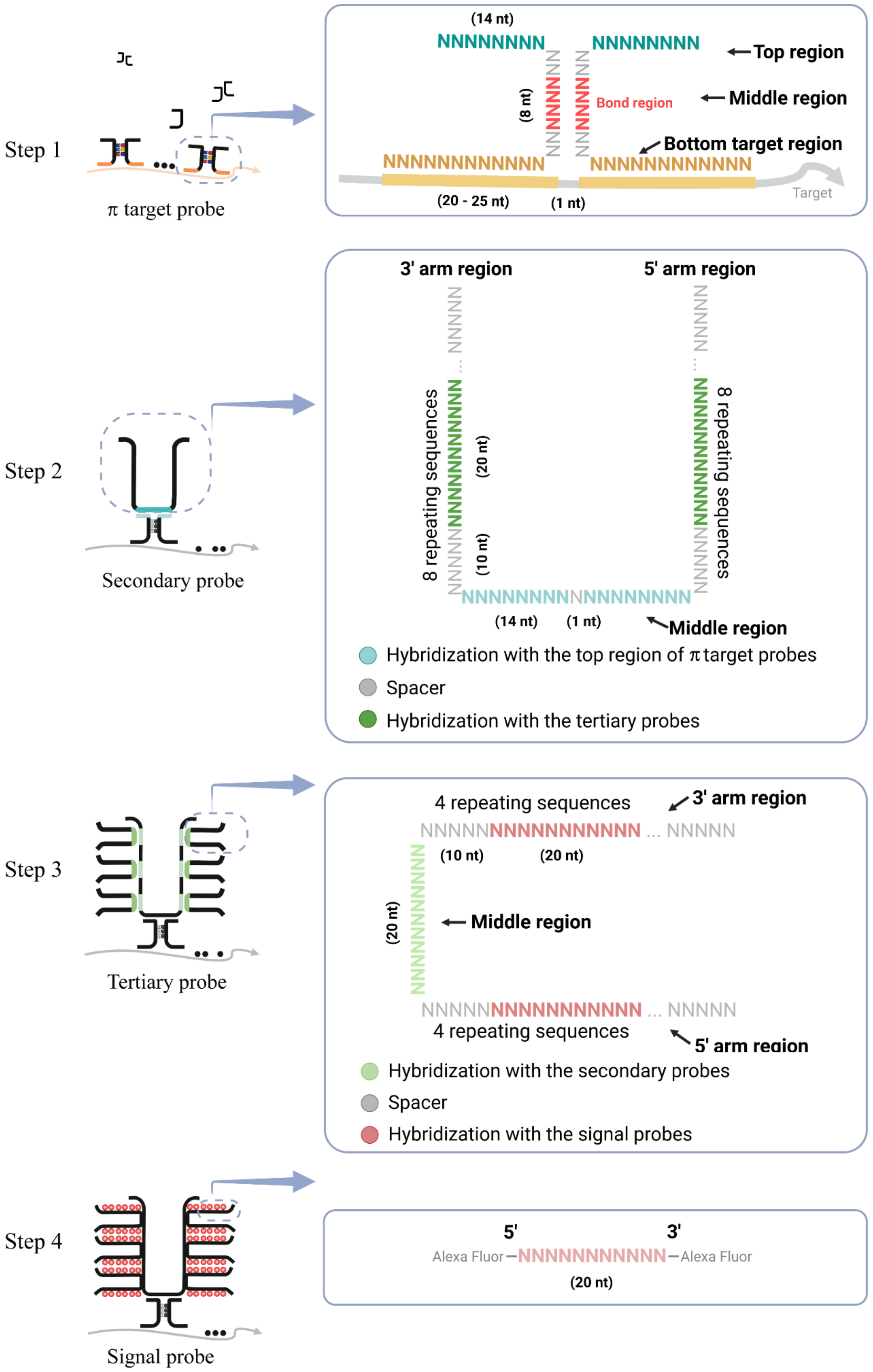

Supplementary Fig. 1 The workflow of π-FISH rainbow and the probe sequence information.

Step 1: Hybridization of π target probes with target genes. The π target probe contains 2–4 complementary base pairs in the middle bond region to form a π shape. Half of the π target probe consists of three sections: bottom target region (20–25 nt), top region (14 nt), and middle region (8 nt). Steps 2 and 3: Hybridization of amplification probes. The total lengths of secondary and tertiary amplification probes are 509 nt and 260 nt, respectively. And both of them consist of two sections: the middle region and the 5′ and 3′ arm regions. Step 4: Hybridization of signal probes with amplification probes. The signal probe is 20 nt and conjugated with fluorophores at both 5′ and 3′ ends.

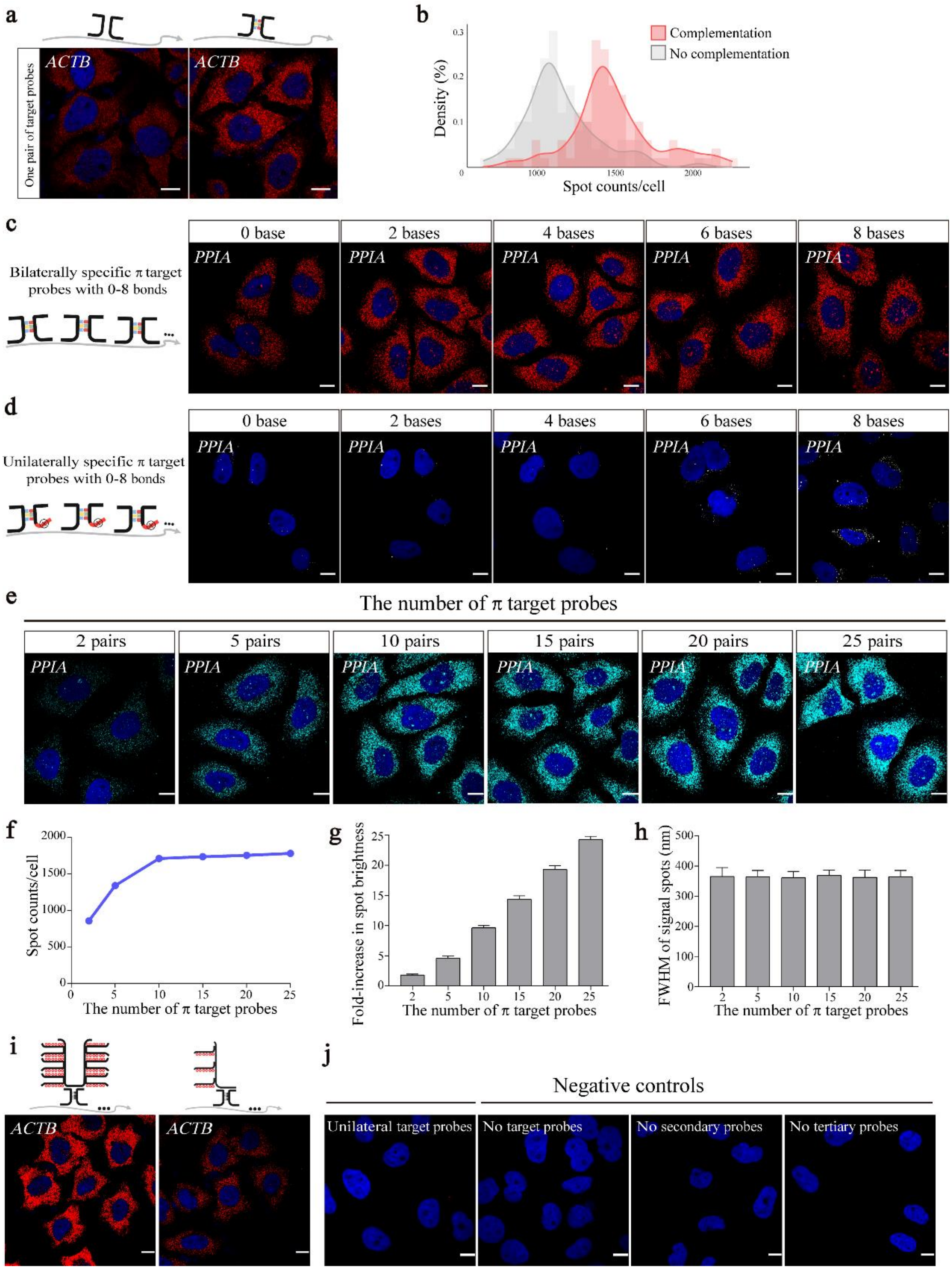

Supplementary Fig. 2 Efficiency and specificity of π-FISH rainbow.

(a) Hybridization efficiency of π target probes (with 2 complementary base pairs; bond region) and traditional split probes (without bond region) in detecting *ACTB* mRNA in HeLa cells using one pair of target probes. Scale bars, 10 μm.

(b) Histogram of spot counts per cell for *ACTB* mRNA detected with one pair of π target probes (with the bond region) and split probes (without bond region). n = 90 cells per group. Source data

are provided as a Source Data file.

(c-d) Effect of different numbers of complementary base pairs (0, 2, 4, 6, and 8) on signal (red) and noise (white). *PPIA* mRNA of HeLa cells was detected with bilaterally specific (c) and unilaterally specific (d) π target probes with different numbers of complementary base pairs. Scale bars, 10 μm.

(e) Detection of *PPIA* mRNA in HeLa cells with different numbers of probe pairs (2, 5, 10, 15, 20, and 25 pairs of π target probes). Scale bars, 10 μm.

(f) Line chart of mean spot counts per cell from (e). n = 30 cells per group. Source data are provided as a Source Data file.

(g) The fold-increase in the brightness of individual *PPIA* mRNA spots in HeLa cells from (e). n = 4000 spots per group. Error bars indicate ± s.e.m. (standard error of the mean). Source data are provided as a Source Data file.

(h) The spot sizes were measured in the hybridization with different numbers of probe pairs (2, 5, 10, 15, 20, and 25 π target probes). The width (full width at half maximum, FWHM) was determined by Gaussian fitting of RNA spots. n = 2500 spots per group. Error bars indicate ± s.e.m. Source data are provided as a Source Data file.

(i) Higher intensity of bilateral U-shaped amplification signal (left) than that of unilateral L-shaped amplification signal (right). Scale bars, 10 μm.

(j) The specificity of π target probes and amplification probes of π-FISH rainbow was validated with multiple negative controls, including unilateral target probes, no target probes, no secondary probes, and no tertiary probes for the hybridization, respectively. Scale bars, 10 μm.

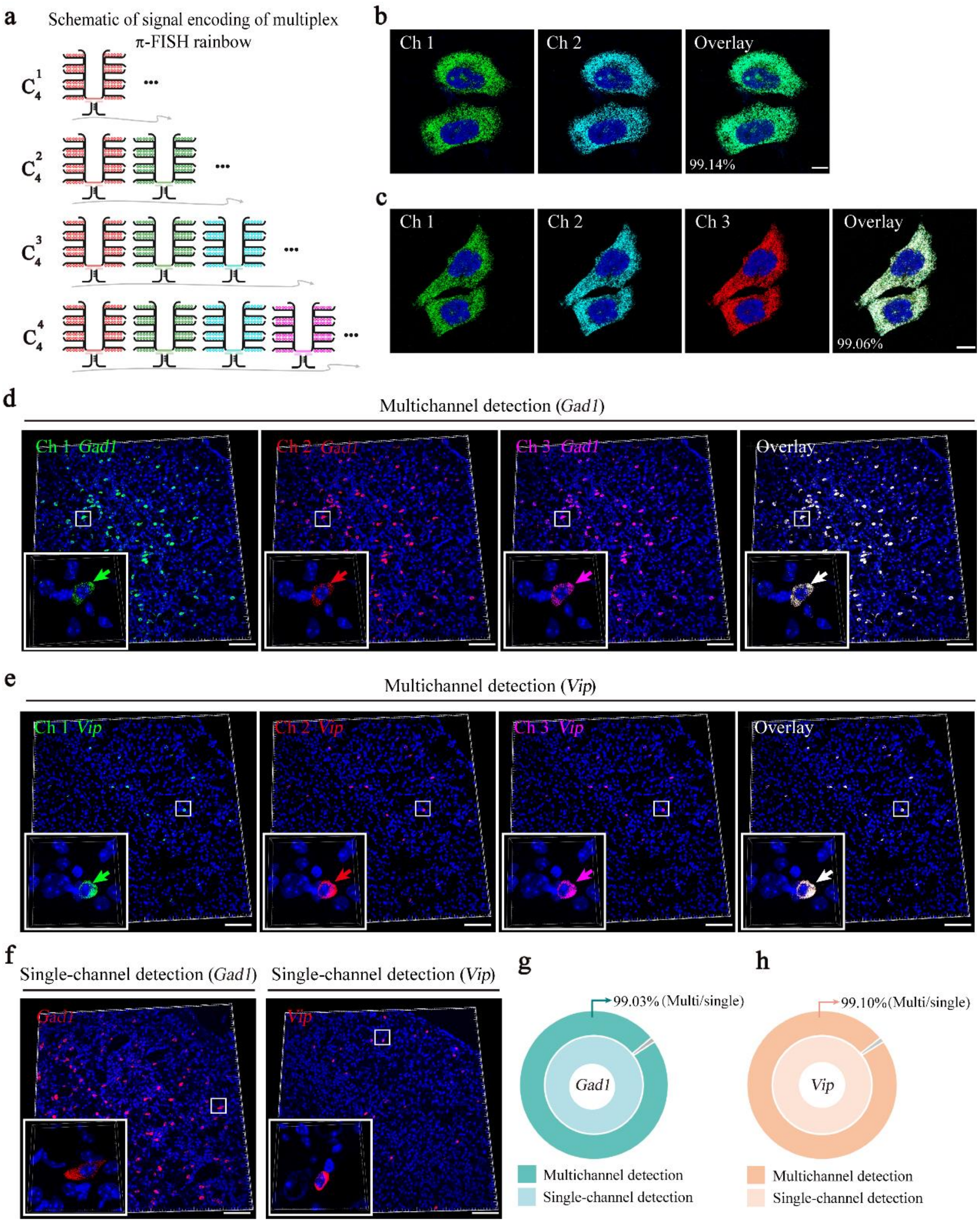

Supplementary Fig. 3 Efficiency and accuracy of π-FISH rainbow in multiplexed detection and decoding.

(a) Schematic of signal coding based on the single or merged colour of double, triple, and quadruple fluorescence channel signal for multiplexed π-FISH rainbow.

(b-c) *In situ* detection of *ACTB* mRNA with two (b) or three (c) fluorescence signal probes. The overlap ratios were 99.14% (b) and 99.06% (c), respectively. n = 50 cells. Ch 1, channel 1; Ch 2, channel 2; Ch 3, channel 3. Scale bars, 10 µm. Source data are provided as a Source Data file.

(d-e) *In situ* detection of *Gad1* (d) and *Vip* (e) genes expression with triple channels in mouse brain tissues. Scale bars, 100 µm.

(f) *Gad1* mRNA (left) and *Vip* mRNA (right) were detected with a single channel in mouse brain tissues, respectively. Scale bars, 100 µm.

(g-h) The efficiency of multichannel detection for *Gad1* (g) and *Vip* (h) from (d-f) were 99.03% and 99.10%, in comparison to single-channel detection, respectively. n = 30 cells. Source data are provided as a Source Data file.

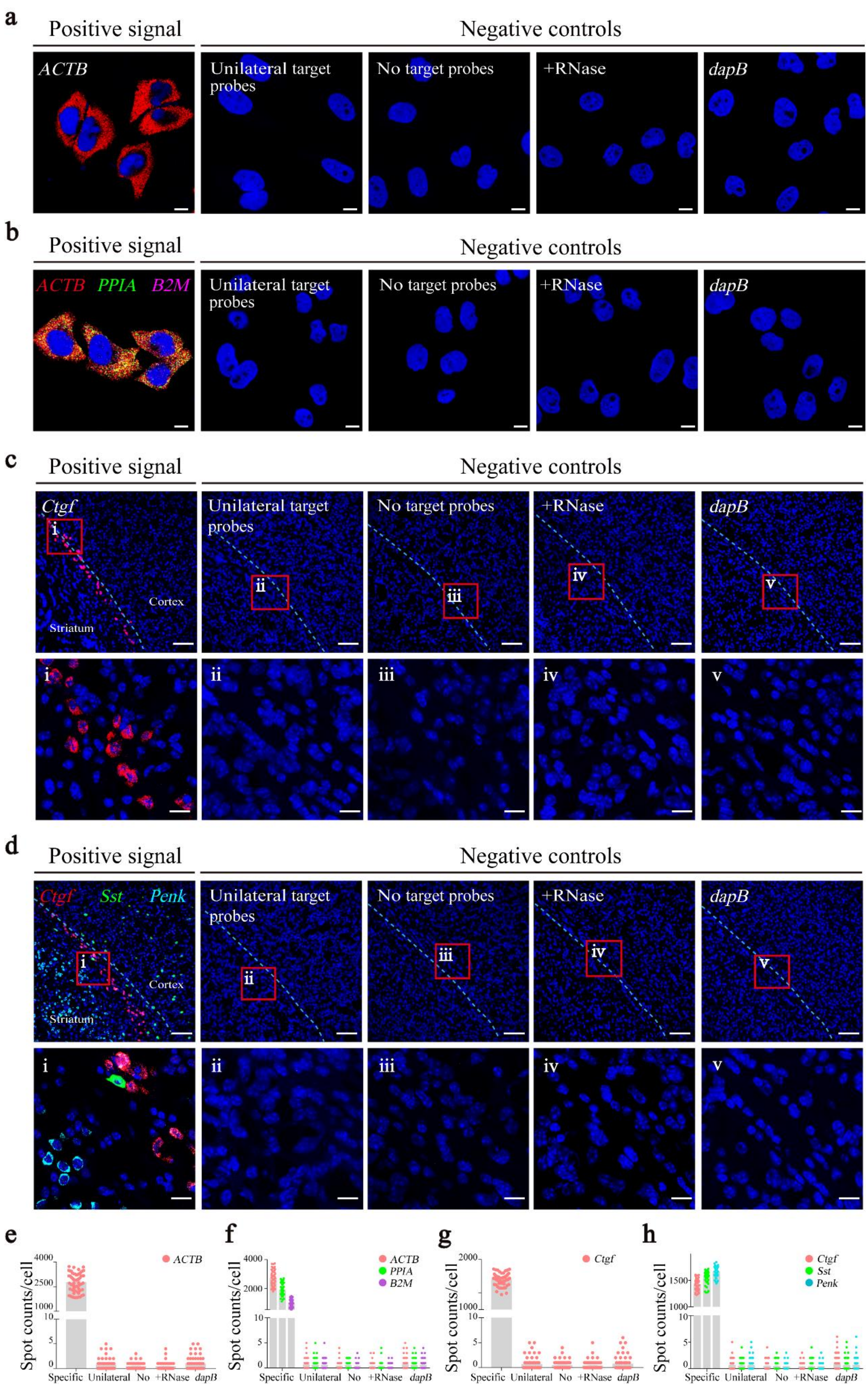

Supplementary Fig. 4 Background of π-FISH rainbow in cells and tissues.

(a-b) The background of π-FISH rainbow in cells was verified by *in situ* detection of *ACTB* (a) and co-detection of *ACTB*, *PPIA*, and *B2M* (b) along with multiple negative controls, including unilateral target probes, no target probes, bilateral target probes with RNase treatment, and target probes of bacterial gene *dapB* in HeLa cells, respectively. Scale bars, 10 µm.

(c-d) The background of π-FISH rainbow in tissues was verified by *in situ* detection of *Ctgf* (c) and co-detection of *Ctgf*, *Penk*, and *Sst* (d) along with multiple negative controls, including unilateral target probes, no target probe, bilateral target probes with RNase treatment, and and target probes of bacterial gene *dapB* in mouse brain sections, respectively. (i-v) Higher magnification of square regions in the upper panels of (c) and (d) were shown in the lower panels. Scale bars, 100 µm (top) and 20 µm (bottom).

(e-h) The histogram of spot counts per cell for positive signals and background noise from (a-d). n = 60 cells per group. Source data are provided as a Source Data file.

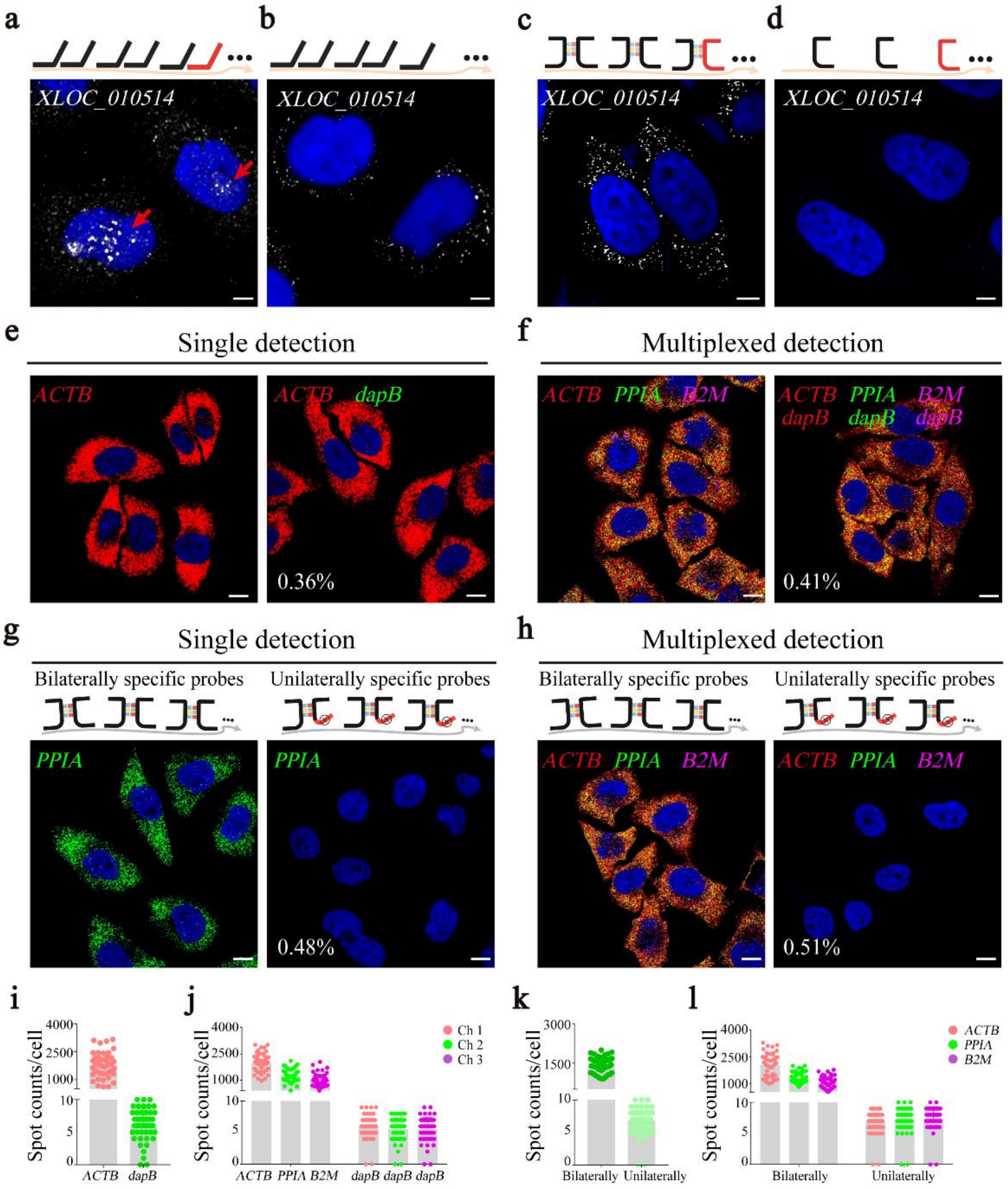

Supplementary Fig. 5 Verification of the false positive rates of π-FISH rainbow.

(a-b) Detection of XLOC_010514 by unamplified FISH. One off-target probe ('rogue' probe, in red) caused false positive signals (red arrows) in the nucleus (a). Removal of the 'rogue' probe eliminated the false positive signals (b). Scale bars, 5 μm.

(c-d) Detection of XLOC_010514 by π-FISH rainbow. No false positive signals were detected despite the 'rogue' probe design when full π target probes (c) or half π target probes (d) were added. Scale bars, 5 μm.

(e-f) False positive rates of single (e) and multiplexed (f) detection for π-FISH rainbow were verified by detecting *ACTB* mRNA and simultaneously detecting *ACTB*, *PPIA*, and *B2M* mRNA, respectively, along with nonspecific probes (bacterial *dapB* probes) addition. The false positive rate

of single detection is the ratio of the total counts of *dapB* over the total spot counts of *ACTB*. The false positive rate of multiplexed detection is the ratio of the false positive spot counts (total spot counts of *ACTB*, *PPIA*, *B2M*, and *dapB* (encoded by three channels) minus the total spot counts of *ACTB*, *PPIA*, and *B2M*) over the total spot counts of *ACTB*, *PPIA*, and *B2M*. Scale bars, 10 μm.

(g-h) The false positive rate of single (g) and multiplexed detection (h) for π-FISH rainbow were further verified by detecting the *PPIA* mRNA alone and simultaneously detecting *ACTB*, *PPIA*, and *B2M* mRNA, with bilaterally and unilaterally specific π target probes. The false positive rate for *PPIA* detection is the total spot counts of *PPIA* with unilaterally specific π target probes over the total spot counts of *PPIA* with bilaterally specific π target probes. The false positive rate of multiplexed detection is the total spot counts of *ACTB*, *PPIA*, and *B2M* with unilaterally specific π target probes over the total spot counts of *ACTB*, *PPIA*, and *B2M* with bilaterally specific π target probes. Scale bars, 10 μm.

(i-l) The histogram of spot/cell for positive signals and background noise from (e-h). Source data are provided as a Source Data file.

The ratios in (e-h) indicated the false positive rates. n = 50 cells per group.

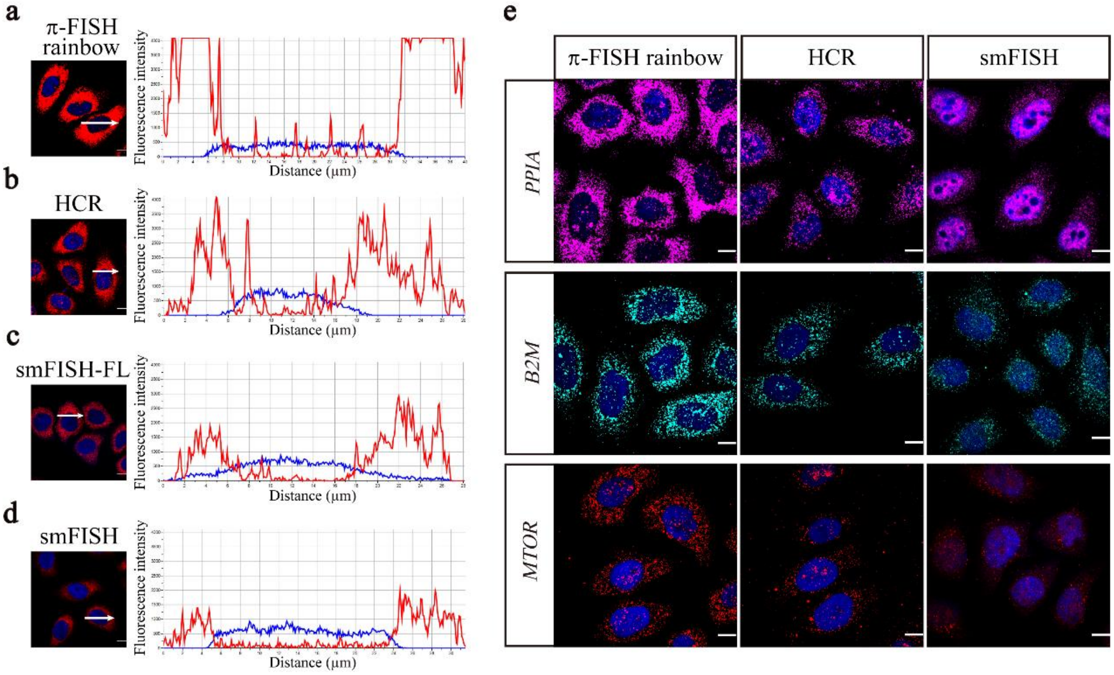

Supplementary Fig. 6 Comparison of hybridization efficiency of π-FISH rainbow with HCR, smFISH, and smFISH-FL.

(a-d) The signal intensity of *ACTB* mRNA was detected by π-FISH rainbow (a), HCR (b), smFISH-FL (c), and smFISH (d) in HeLa cells, respectively. Equal concentrations of target probes with equal lengths were used for all methods except smFISH-FL, where the probes targeted the whole transcript. As illustrated, the straight lines were drawn across the cells, of which the intensity profiles were plotted (*ACTB* mRNA, red curve lines; DAPI, blue curve lines). Scale bars, 10 μm.

(e) Detection of *PPIA, B2M*, and *MTOR* mRNA by π-FISH rainbow, HCR, and smFISH, respectively. Scale bars, 10 μm.

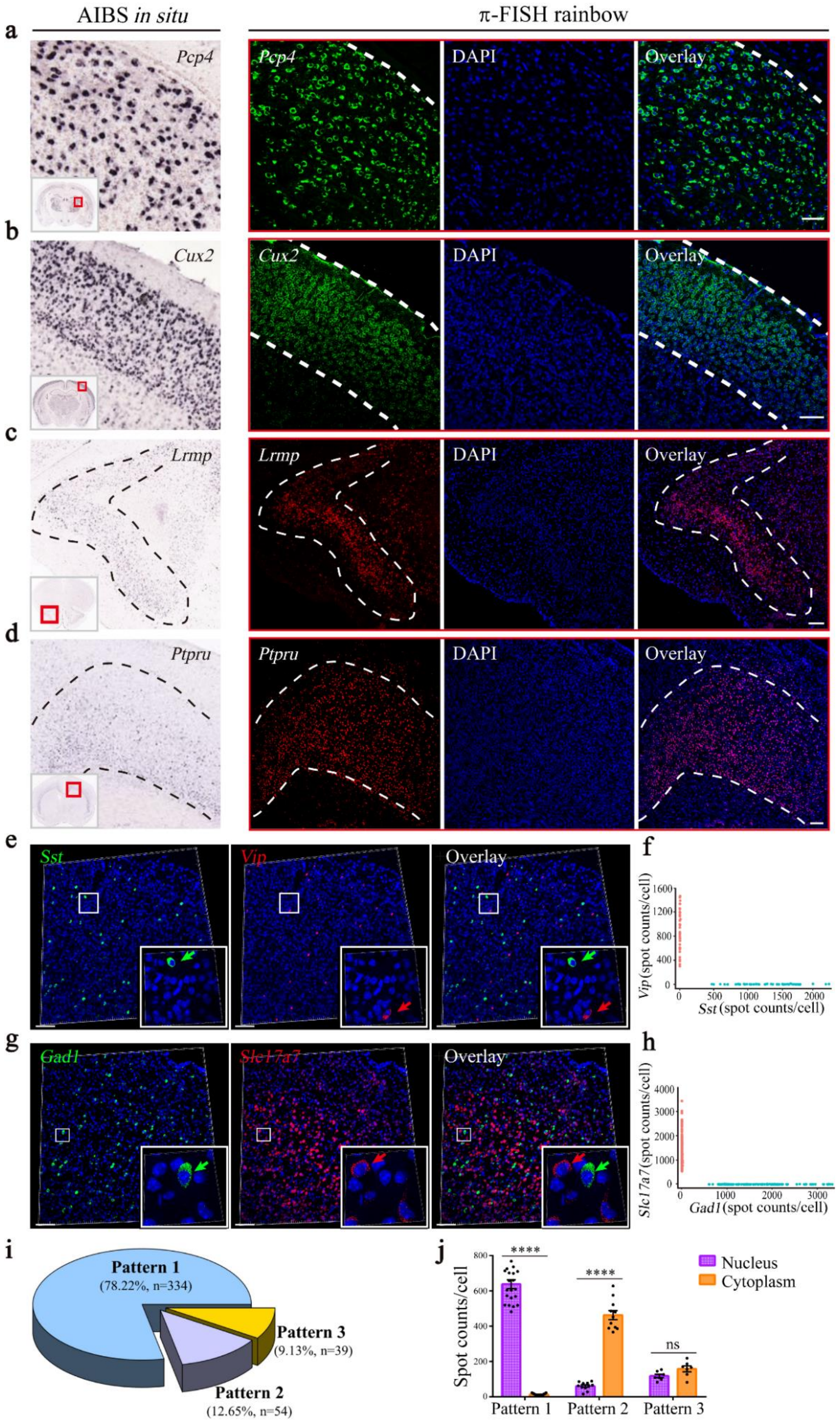

Supplementary Fig. 7 Faithful *in situ* presentation of gene expression by π-FISH rainbow in cells and tissues.

(a-d) The expression patterns of highly expressed genes *Cux2* (a) and *Pcp4* (b), as well as lowly expressed genes *Lrmp* (c) and *Ptpru* (d) in mouse brain detected by π-FISH rainbow, were consistent with that described by the Allen Institute of Brain Science (AIBS). Scale bars, 50 μm (a and b); 100 μm (c and d).

(e) Co-detection of *Sst* and *Vip,* two mutually exclusively expressing genes in different subclasses of interneurons in mouse cerebral cortex, by π-FISH rainbow. The green and red arrows indicated Sst^{+} and Vip^{+} neurons, respectively. Scale bars, 100 μm.

(f) The scatter plot of spot counts per cell for mutually exclusive genes *Vip* and *Sst* from (e). n = 89 cells. Source data are provided as a Source Data file.

(g) Co-detection of inhibitory (*Gad1*) and excitatory neuron marker (*Slc17a7*) in the mouse cerebral cortex by π-FISH rainbow. The green and red arrows indicated $Gad1^{+}$ and $Slc17a7^{+}$ neurons, respectively. Scale bars, 100 μm.

(h) The scatter plot of spot counts per cell for mutually exclusive genes *Slc17a7 and Gad1* from (g). n = 848 cells. Source data are provided as a Source Data file.

(i) The percentages of three subcellular localization patterns for *MALAT1* mRNA were 78.2% (nucleus), 12.7% (cytoplasm), and 9.1% (low expression but uniform location in both cytoplasm and nucleus), respectively.

(j) Histogram of spot counts per cell for three subcellular localization patterns of *MALAT1* mRNA detected by π-FISH rainbow. n = 39 cells. Data were expressed as mean ± s.e.m. Two-tailed unpaired *t* test was used to compare the two groups. Pattern 1, P = 2.54E-26; Pattern 2, P = 1.86E-12; Pattern 3, P = 0.054. ****$P < 0.0001$; ns, not significant. Source data are provided as a Source Data file.

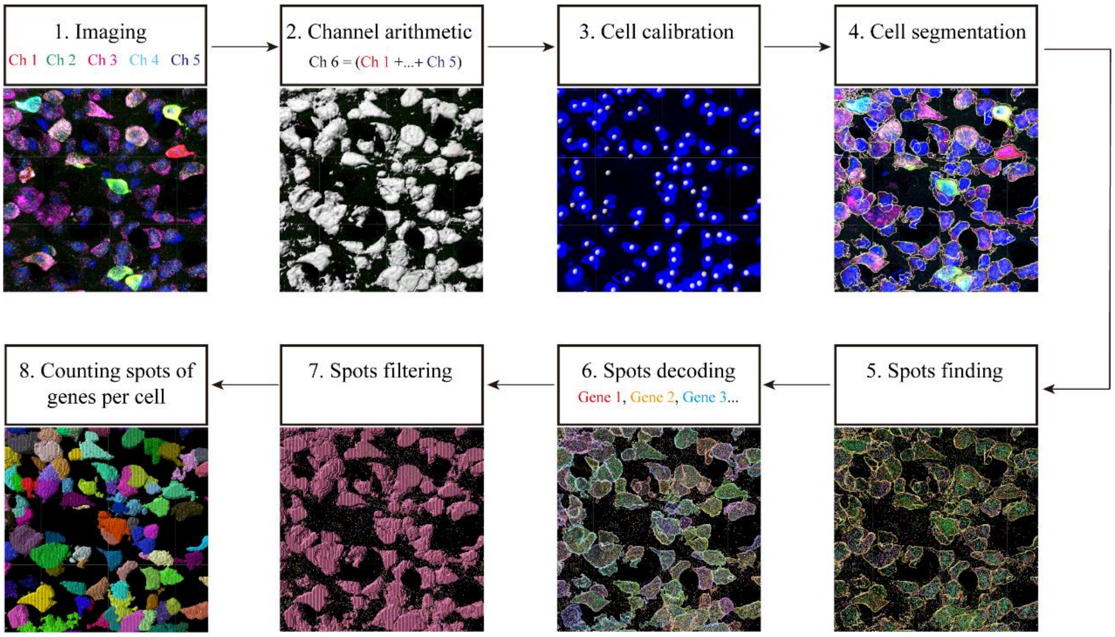

Supplementary Fig. 8 Image analysis pipeline for multiplexed π-FISH rainbow.

Diagram of image analysis pipeline for multiplexed π-FISH rainbow. (1) Imaging: Raw imaging data were obtained from five fluorescence channels. (2) Channel arithmetic: The boundary of signals per cell was accurately determined using five-channel arithmetic based on gene expression signals. (3) Cell calibration: Cell numbers were calibrated according to the nuclear DAPI signal. (4) Cell segmentation: Each cell was segmented according to channel arithmetic and cell number. (5) Spots finding: Each spot was automatically identified based on the fluorescence channel. (6) Spots decoding: Each spot was decoded into its corresponding gene based on channel combinations. (7) Spots filtering: Only spot signals within the boundary of cell segmentation were labelled and locked to prevent spot interference outside the boundary. (8) Counting spots of genes per cell.

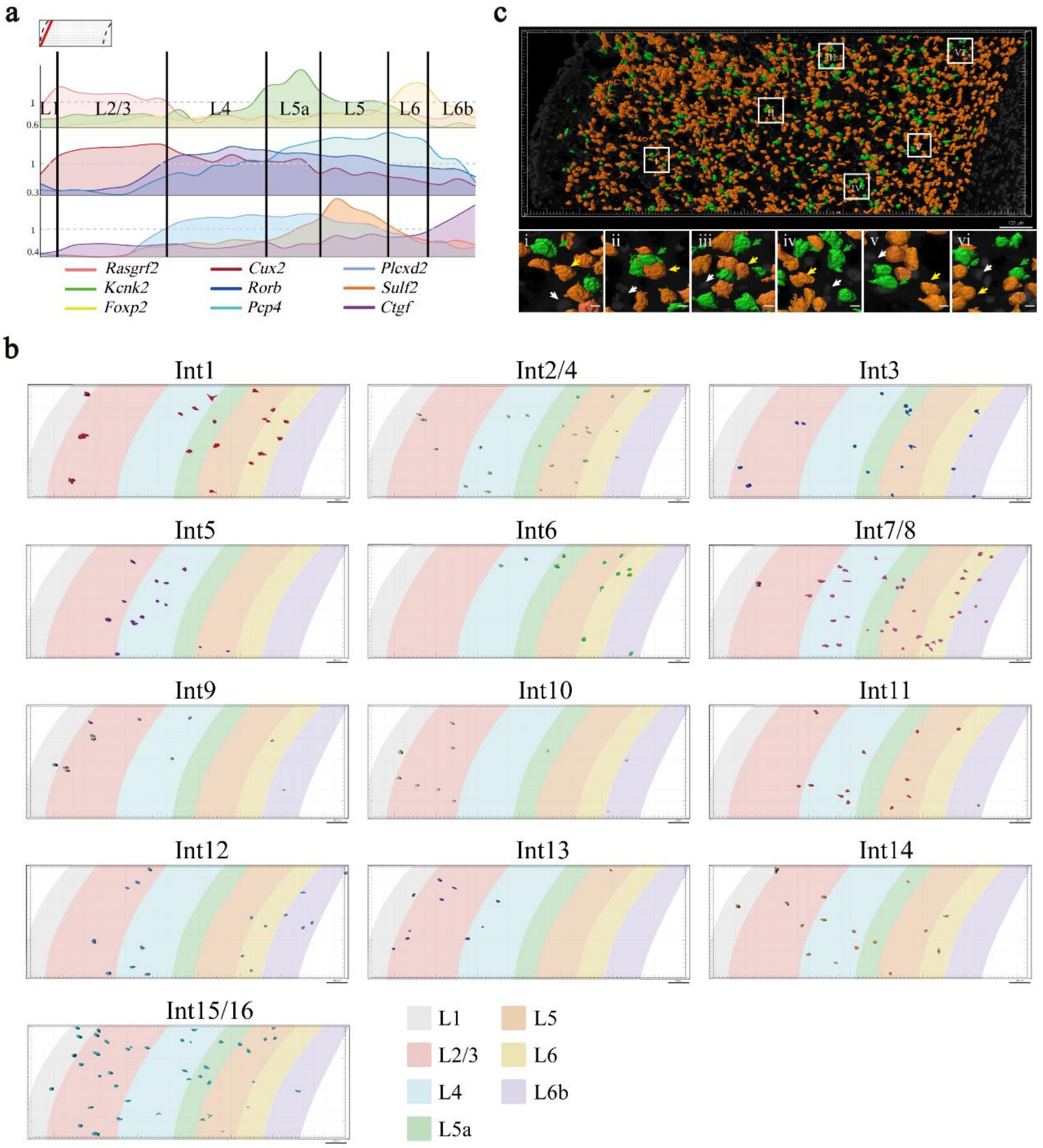

Supplementary Fig. 9 Signal decoding of two-round hybridization by π-FISH rainbow.

(a) Spatial histograms of nine marker gene signals from (Fig. 2c) across the S1 cortex. Each gene has a peak wave distribution in its main expression layer. The ordinate value indicated the ratio of real signal intensity over mean signal intensity for nine marker genes along the whole cerebral cortex.

(b) Layer distribution of 13 *Gad1*$^{+}$ interneuron subclasses in S1 based on the first-round layer-specific marker gene hybridization information. Int1 to Int16 indicate the interneuron subclasses defined by scRNA-seq from the publication by Zeisel *et al.*, 2015[5]. Scale bars, 100 μm.

(c) Representative cell type-resolved spatial map in the S1 cortex: Cells were marked as three different types: excitatory neurons (orange, yellow arrow indicated), inhibitory neurons (green, green arrow indicated), and non-neuronal cells (grey, white arrow indicated). (i-vi) Higher-

resolution comparison images of the cells were listed at the bottom. Scale bars, 100 μm (top) and 5 μm (bottom).

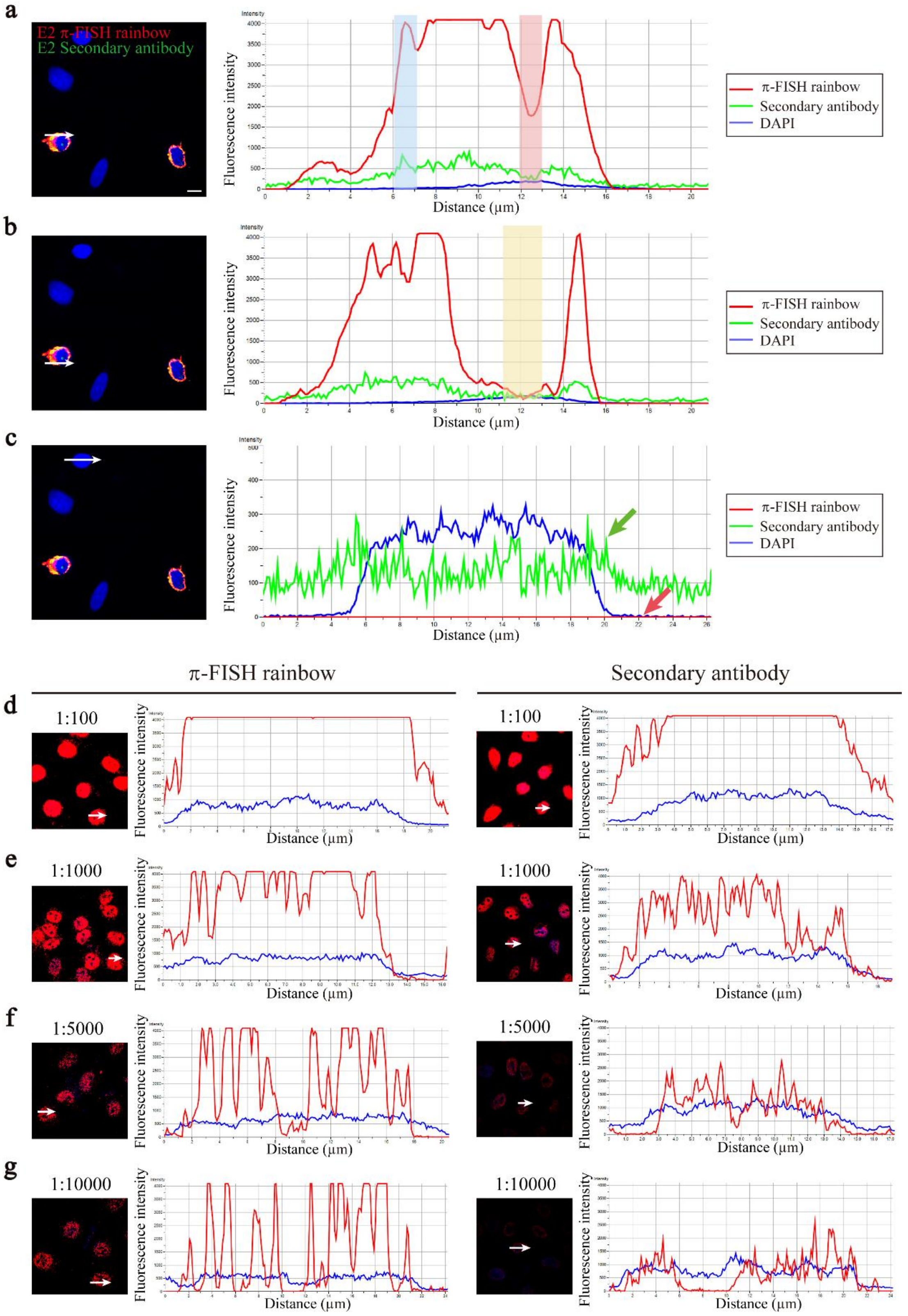

Supplementary Fig. 10 Comparison of intensity profiles and specificity between π-FISH rainbow and conventional immunostaining for protein detection.

(a-b) Intensity profiles (right) along the straight lines on top (a) and bottom (b) of the whole cell (left). Both π-FISH rainbow and conventional immunostaining showed similar signal patterns and

signal variation trends (indicated by blue, red, and yellow rectangular frames) in E2-positive cells. However, the signal generated by π-FISH rainbow was much higher than that of the immunostaining. Scale bar, 10 μm.

(c) Intensity profiles along the lines across the whole cell lacking E2 for secondary antibody (green arrow), π-FISH rainbow (red arrow), and DAPI fluorescence, respectively.

(d-g) Intensity profiles along straight lines crossing the whole cell were illustrated for comparisons of signal sensitivity and amplification capabilities between π-FISH rainbow and conventional immunostaining with primary antibodies (pol II) at serial dilutions of 1:100 (d), 1:1000 (e), 1:5000 (f), and 1:10,000 (g).

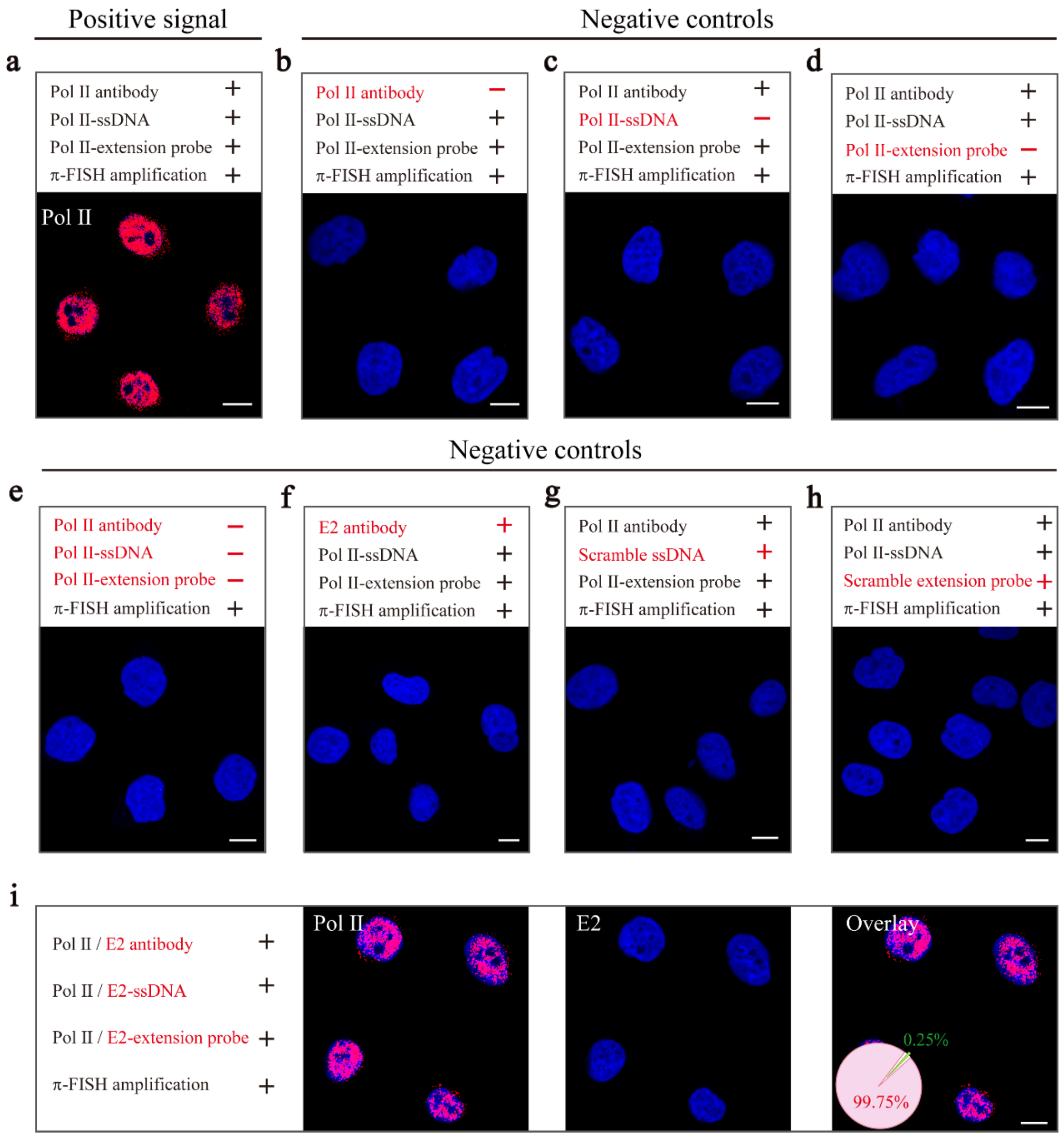

Supplementary Fig. 11 The specificity and efficiency of π-FISH rainbow in protein detection.

(a) Detection of pol II protein in Hela cells by π-FISH rainbow using ssDNA conjugated pol II antibody and corresponding extension probe. Scale bar, 10 μm.

(b-d) Detection of pol II protein in Hela cells by π-FISH rainbow without pol II antibody (b), specific ssDNA (c), and specific extension probe (d), respectively. Scale bars, 10 μm.

(e) Detection of pol II protein in Hela cells by π-FISH rainbow without antibody, ssDNA, and extension probe. Scale bar, 10 μm.

(f-h) Detection of pol II protein in Hela cells (without E2 protein) with E2 antibody (f), scramble ssDNA (g), and scramble extension probe (h), respectively. Scale bars, 10 μm.

(i) The off-target and false-positive rate was verified by co-detection of pol II protein (red) and

CSFV E2 protein (green) by π-FISH rainbow in Hela cells. The percentage of false-positive rate of π-FISH rainbow in protein detection was 0.25%. n = 30 cells. Scale bar, 10 μm. Source data are provided as a Source Data file.

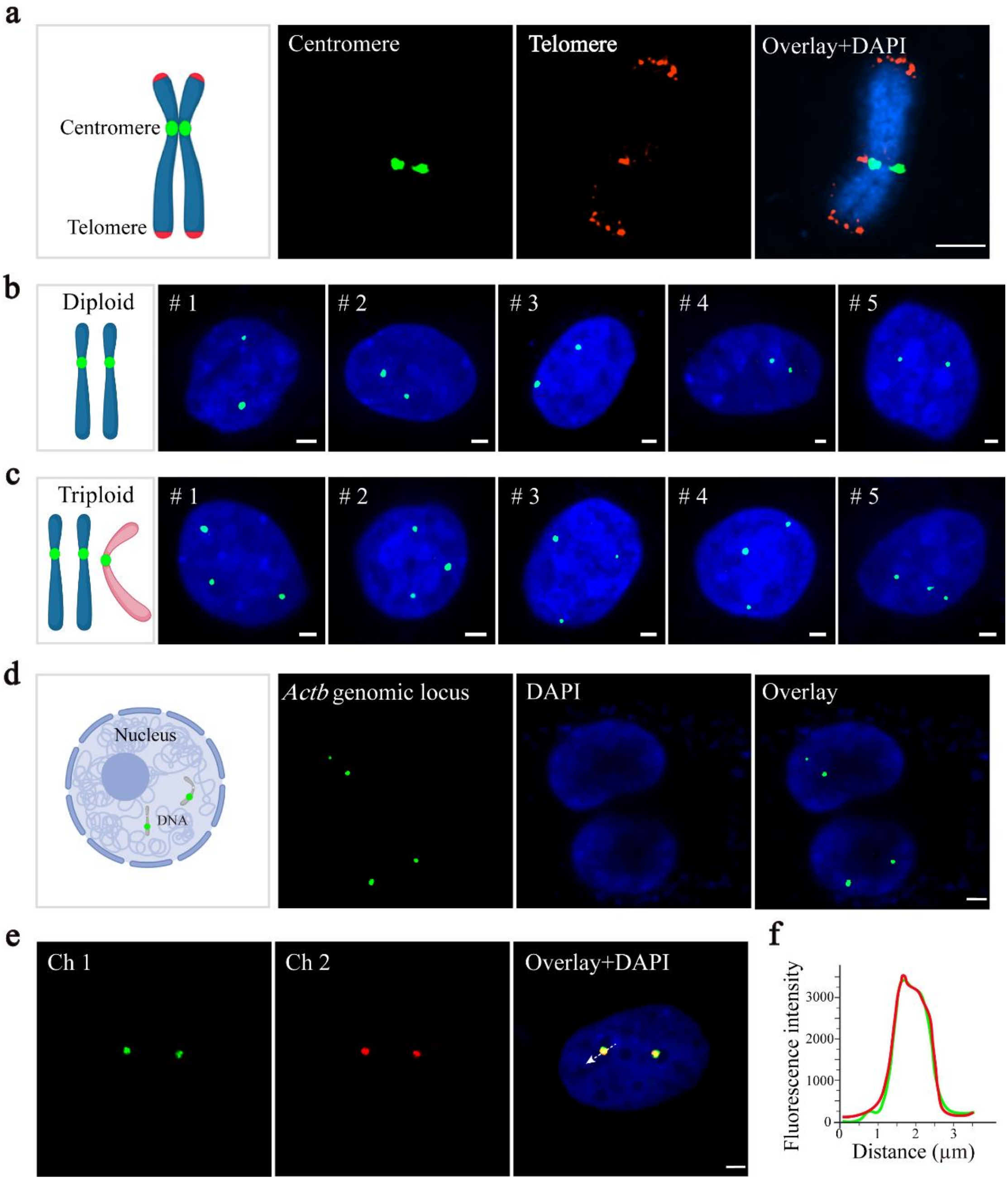

Supplementary Fig. 12 *in situ* detection of DNA by π-FISH rainbow.

(a) Diagram (left) and π-FISH rainbow detection (right) of repetitive genome regions from centromeres (green) and telomeres (red) in chromosome 2 of HeLa cells. Scale bars, 2.5 µm.

(b-c) Diagram (left) and π-FISH rainbow detection (right) of diploid (b) and triploid (c) chromosome 2 in HeLa cells. Scale bars, 2.5 µm. n = 89 cells.

(d) Diagram (left) and detection of *Actb* genomic locus in BHK cells using π-FISH rainbow with 35 pairs of π target probes. Scale bar, 2.5 µm.

(e) Merged signals for *Actb* genomic loci in BHK cells using two fluorescent signal probes. Scale

bar, 2.5 µm.

(f) Intensity profiles of two fluorescent signals for the signal spot (white arrow in e). The signal probes were labelled with Alexa Fluor 488 (green) and Alexa Fluor 594 (red).

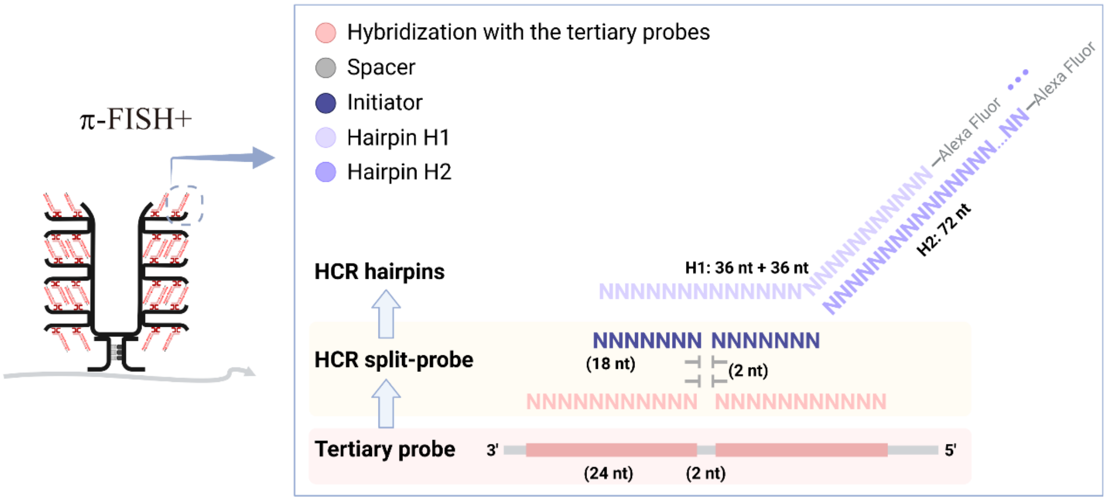

Supplementary Fig. 13 Schematic overview and probe sequence information for π-FISH+. For π-FISH+, the sequence information and hybridization procedure of steps 1–3 are the same as in π-FISH rainbow, while the signal probe in step 4 of π-FISH rainbow is replaced with the HCR split-probe and self-folding hairpins to further amplify the signal according to HCR 3.0. Each tertiary amplification probe can combine four HCR amplification reactions in this process. The HCR split-probe consists of left and right parts. Half of the split-probe consists of three sections: bottom target region (24 nt), top region (18 nt), and middle region (2 nt). The top regions on the left and right sides of the HCR split-probe constitute initiator for HCR and form a complementary sequence to hairpin H1. The signal probes consist of hairpin H1 and H2 with 72 nt for each.

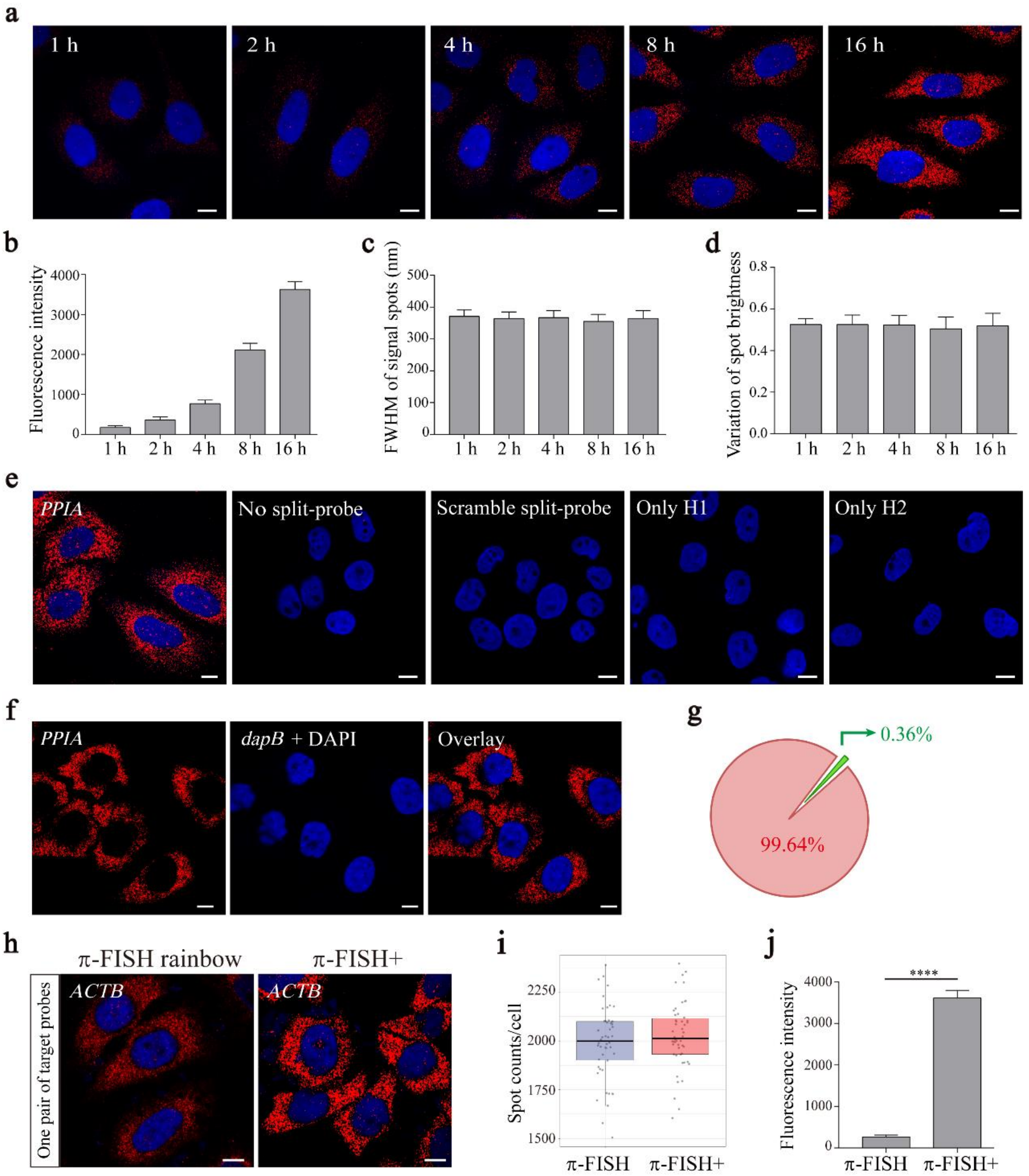

Supplementary Fig. 14 Validation of efficiency, specificity, and tunability of π-FISH+ strategy.

(a-d) Detection of *PPIA* mRNA in HeLa cells with one pair of π target probes by π-FISH+, in which the HCR amplification was performed for 1 h, 2 h, 4 h, 8 h, and 16 h, respectively (a). The fluorescence intensity of individual spots (b), spot sizes (c), and variation coefficients of spot brightness (d) were measured. The width (FWHM) was determined by the Gaussian fitting of RNA spots. n = 2000 spots per group. Data were expressed as mean ± s.e.m. Scale bars, 10 μm. Source data are provided as a Source Data file.

(e) Detection of *PPIA* mRNA in HeLa cells by π-FISH+ with specific HCR split-probe, without

HCR split-probe, with HCR split-probe containing scramble initiator, and with hairpin H1 or H2 only, respectively. Scale bars, 10 μm.

(f-g) The false positive rate of HCR amplification in π-FISH+ was verified by co-detection of *PPIA* (red) and *dapB* (nonspecific binding, green) in HeLa cells (f), and the false positive rate was 0.36% (g). Scale bars, 10 μm. n = 30 cells. Source data are provided as a Source Data file.

(h) Detection of *ACTB* mRNA in HeLa cells with one pair of π target probes by π-FISH rainbow (left) and π-FISH+ (right), respectively. Scale bars, 10 μm.

(i) The spot counts per cell of *ACTB* mRNA detected by π-FISH+ with one pair of π target probes were comparable to that by π-FISH. n =50 cells per group. Box-plot with midline = median, box limits = Q1 (25th percentile)/Q3 (75th percentile), whiskers = minimum and maximum values, points = outliers (>1.5 interquartile range). Source data are provided as a Source Data file.

(j) The fluorescence intensity of *ACTB* mRNA detected by π-FISH+ with one pair of π target probes was significantly higher than that of π-FISH. $P = 5.83E\text{-}306$. ****$P < 0.0001$. n = 2000 spots per group. Error bars indicate ± s.e.m. Two-tailed unpaired *t* test was used to compare the two groups. Source data are provided as a Source Data file.

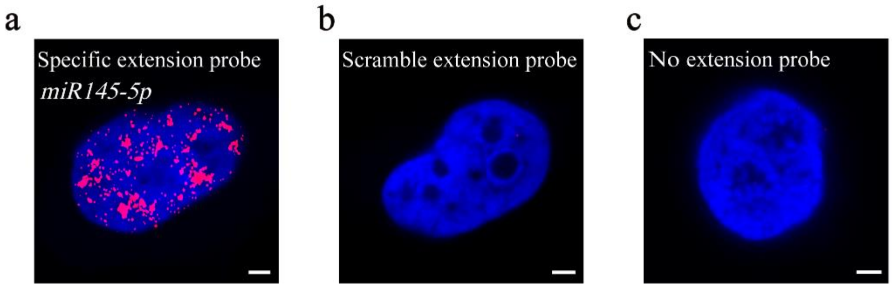

Supplementary Fig. 15 Validation of the specificity and efficiency of π-FISH+ for miRNA detection.

(a) The *miR145-5p* was detected using a specific extension probe as a positive control. Scale bar, 2.5 μm.

(b-c) The specificity of the miRNA complementary sequence within the extension probe was verified with a scramble extension probe (nonspecific binding, b) and no extension probe (c) for the hybridization. Scale bars, 2.5 μm.

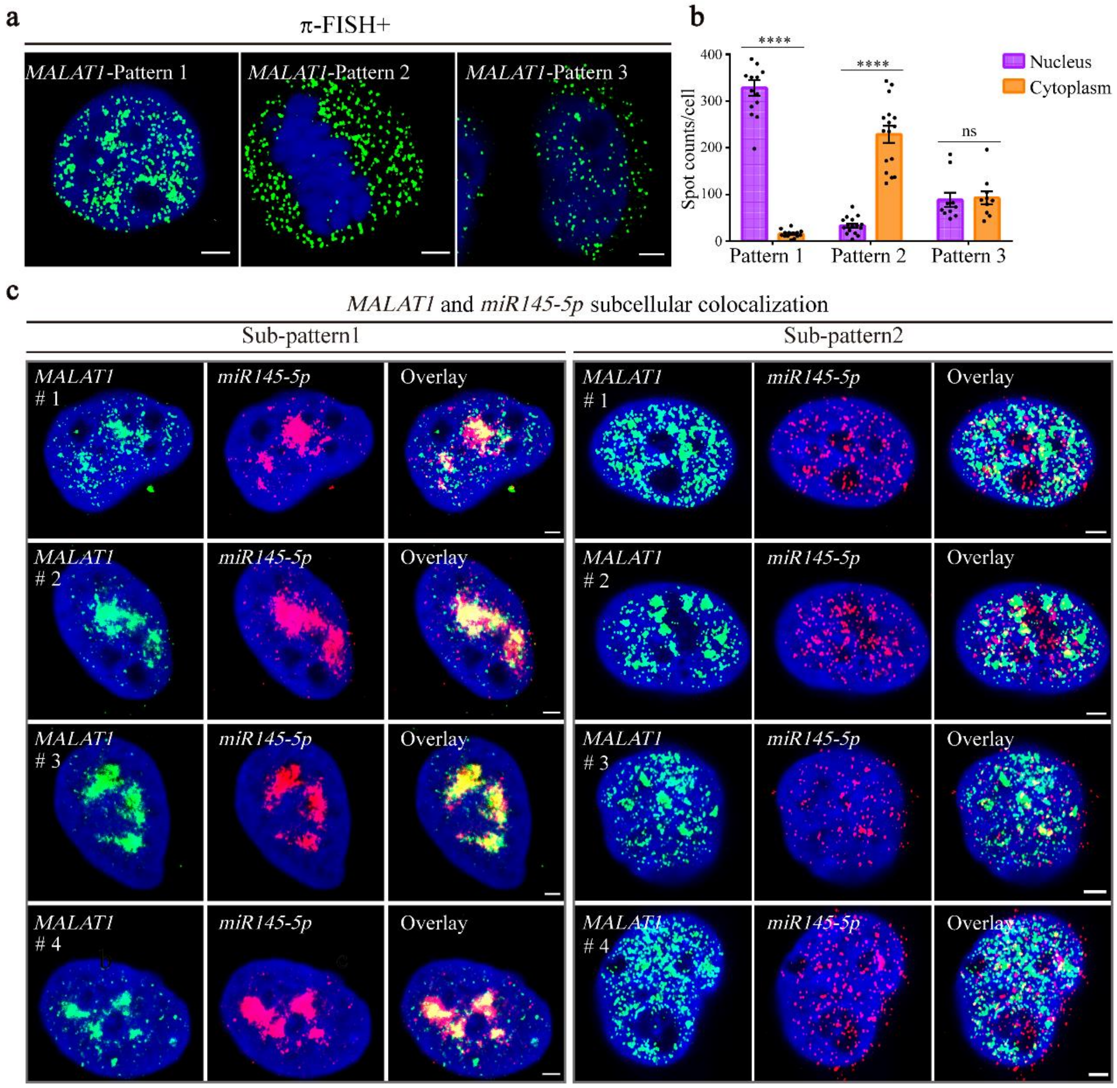

Supplementary Fig. 16 Co-detection of *miR145-5p* and *MALAT1* by π-FISH+.

(a) Consistent with the π-FISH rainbow results, π-FISH+ identified three distinct subcellular localization patterns of *MALAT1*: mainly in the nucleus, mainly in the cytoplasm, and evenly distributed in both the nucleus and cytoplasm. Scale bars, 2.5 μm.

(b) Histogram of spot counts per cell for three subcellular localization patterns of *MALAT1* detected by π-FISH+. n = 40 cells. Data were expressed as mean ± s.e.m. Two-tailed unpaired *t* test was used to compare the spot counts between nucleus and cytoplasm. Pattern 1, $P < 1\times10^{-15}$; Pattern 2, $P = 9.01\times10^{-9}$; Pattern 3, $P = 0.83$). ****$P < 0.0001$; ns, not significant. Source data are provided as a Source Data file.

(c) The *miR145-5p* and its sponge LncRNA *MALAT1* were co-detected by π-FISH+. Two sub-patterns: some cells show a more diffuse distribution of both signals in the nucleus, while others show larger aggregated signals. Scale bars, 2.5 μm.

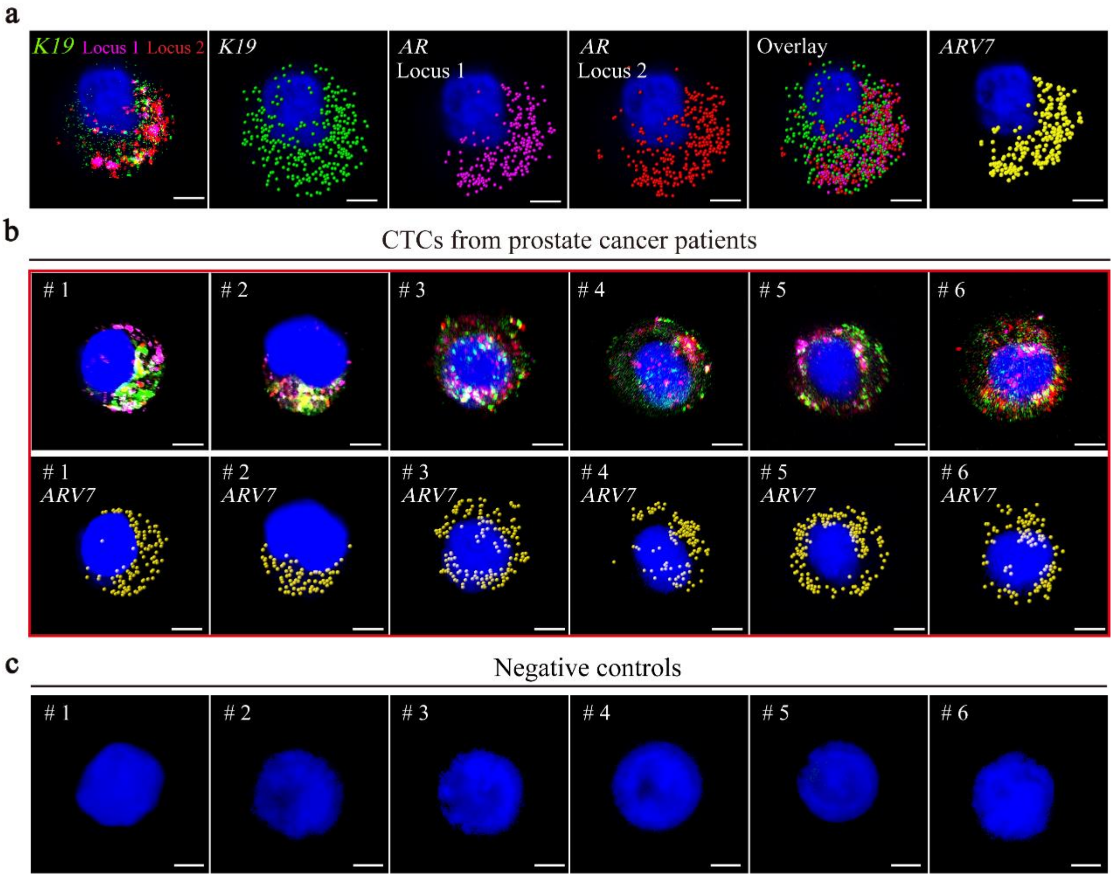

Supplementary Fig. 17 Detection of *ARV7* splicing variant in circulating tumour cells from prostate cancer patients.

(a) Detection and decoding of *K19*, *ARV7* locus 1, and *ARV7* locus 2. *ARV7* was identified by simultaneously labelling *ARV7* locus 1 (transcript in *ARV1*, *ARV2*, *ARV4*, and *ARV7*) and locus 2 (transcript in *ARV5* and *ARV7*) using two fluorescence signal probes. The merged signal (yellow) of locus 1 (magenta) and locus 2 (red) represent *ARV7* signals, as well as the *K19* signal (green) as a CTCs biomarker. Scale bars, 5 µm.

(b) Application of π-FISH+ for *ARV7* detection in multiple CTCs from two more prostate cancer patients. Scale bars, 5 µm.

(c) Healthy human blood cells were used as negative controls. Scale bars, 5 µm.
