# Supplementary material for: Highly efficient and robust π-FISH rainbow for multiplexed in situ detection of diverse biomolecules
Source: Nat Commun. 2023 Jan 27;14:443. doi: 10.1038/s41467-023-36137-4 (PMC9883232; doi:10.1038/s41467-023-36137-4)
Supplement: Supplementary file 3 — Description of Additional Supplementary Files [file 41467_2023_36137_MOESM3_ESM.pdf]

Title: Supplementary Data 1:

Description: This is a Microsoft Excel file containing Target probes, Amplification and signal probes, and HCR signal probes as separate tabs. Target probes contain all target probe sequences of π-FISH rainbow, π-FISH+, smFISH, smFISH-FL, and HCR. Amplification and signal probes contain all the sequences of the secondary amplification probes, tertiary amplification probes, and signal probes of π-FISH rainbow and π-FISH+. HCR signal probes contain all hairpin sequences of HCR.
