# Supplementary material for: Highly efficient and robust π-FISH rainbow for multiplexed in situ detection of diverse biomolecules
Source: Nat Commun. 2023 Jan 27;14:443. doi: 10.1038/s41467-023-36137-4 (PMC9883232; doi:10.1038/s41467-023-36137-4)
Supplement: Supplementary file 5 — Reporting Summary [file 41467_2023_36137_MOESM5_ESM.pdf]

nature portfolio

Corresponding author(s): Jinxia Dai and Gang Cao

Last updated by author(s): Jan 18, 2023

Reporting Summary

Nature Portfolio wishes to improve the reproducibility of the work that we publish. This form provides structure for consistency and transparency in reporting. For further information on Nature Portfolio policies, see our Editorial Policies and the Editorial Policy Checklist.

Statistics

For all statistical analyses, confirm that the following items are present in the figure legend, table legend, main text, or Methods section.

n/a	Confirmed	
☐	☒	The exact sample size (*n*) for each experimental group/condition, given as a discrete number and unit of measurement
☐	☒	A statement on whether measurements were taken from distinct samples or whether the same sample was measured repeatedly
☐	☒	The statistical test(s) used AND whether they are one- or two-sided<br>*Only common tests should be described solely by name; describe more complex techniques in the Methods section.*
☒	☐	A description of all covariates tested
☐	☒	A description of any assumptions or corrections, such as tests of normality and adjustment for multiple comparisons
☐	☒	A full description of the statistical parameters including central tendency (e.g. means) or other basic estimates (e.g. regression coefficient) AND variation (e.g. standard deviation) or associated estimates of uncertainty (e.g. confidence intervals)
☐	☒	For null hypothesis testing, the test statistic (e.g. *F*, *t*, *r*) with confidence intervals, effect sizes, degrees of freedom and *P* value noted<br>*Give P values as exact values whenever suitable.*
☒	☐	For Bayesian analysis, information on the choice of priors and Markov chain Monte Carlo settings
☒	☐	For hierarchical and complex designs, identification of the appropriate level for tests and full reporting of outcomes
☒	☐	Estimates of effect sizes (e.g. Cohen's *d*, Pearson's *r*), indicating how they were calculated

Our web collection on statistics for biologists contains articles on many of the points above.

Software and code

Policy information about availability of computer code

Data collection	Leica TCS SP8 STED softwere (LAS X ver 3.5.5.19976), Nikon N-SIM softwere (NIS ElementsAR ver. 4.50.00), and Nikon N-STORM softwere (NIS ElementsAR ver. 5.30.02) were used to acquire images.
Data analysis	Nikon N-STORM softwere (NIS ElementsAR ver. 5.30.02); Bitplane Imaris (Version 9.9); Graphpad Prism (version 9); MATLAB (R2018B).

For manuscripts utilizing custom algorithms or software that are central to the research but not yet described in published literature, software must be made available to editors and reviewers. We strongly encourage code deposition in a community repository (e.g. GitHub). See the Nature Portfolio guidelines for submitting code & software for further information.

Data

Policy information about availability of data

All manuscripts must include a data availability statement. This statement should provide the following information, where applicable:

- Accession codes, unique identifiers, or web links for publicly available datasets
- A description of any restrictions on data availability
- For clinical datasets or third party data, please ensure that the statement adheres to our policy

The authors declare that the data supporting the findings of this study are available within the article, Supplementary information, and Source data file. The sequence information of probes in this study is provided in the Supplementary information. The raw data for all Figures and Supplementary Figures are provided in the Source data file. Source data are provided with this paper.

Human research participants

Policy information about studies involving human research participants and Sex and Gender in Research.

Reporting on sex and gender	In this study, we detected anti-androgen therapy-resistant marker ARV7 splicing variants in circulating tumour cells from male patients.
Population characteristics	The human blood samples were collected from three male prostate cancer patients (58-65 years old). We had informed consent from the patients.
Recruitment	The blood samples of prostate cancer patients were gifted by Department of Blood Transfusion, Wuhan hospital of Traditional Chinese and Western Medicine, Huazhong University of Science and Technology.
Ethics oversight	The use of human blood samples was approved by the Scientific Ethic Committee of Huazhong Agricultural University (202204060001).

Note that full information on the approval of the study protocol must also be provided in the manuscript.

Field-specific reporting

Please select the one below that is the best fit for your research. If you are not sure, read the appropriate sections before making your selection.

☒ Life sciences ☐ Behavioural & social sciences ☐ Ecological, evolutionary & environmental sciences

For a reference copy of the document with all sections, see nature.com/documents/nr-reporting-summary-flat.pdf

Life sciences study design

All studies must disclose on these points even when the disclosure is negative.

Sample size	No statistical methods were used to pre-determine the sample size. Each experiment was replicated at least three times. For mapping the spatial cell types of the mouse cerebral cortex, brain slices were acquired from at least three mice (three slices per mouse). For the applications of our method in different organisms (microorganisms, plants, and animals) and different samples (whole-mount and tissue sections), at least 10 zebrafish whole-mount samples, 8 slices for a total of five maize ears, 10 brain slices from 3 mice brain, as well as tens of ASFV infected PAMs, M. tuberculosis, and T. gondii infected HFF cells were subjected for the experiments. For cell samples (HEK293T, HeLa, BHK, A549, LNCaP, PC3, PK-15), images were acquired from 30-60 cells for each experiment. We believe the sample size is sufficient to verify the performance of our method since all results were reliably reproduced. The precise number of cell and tissue sections is reported in the figure legends and method.
Data exclusions	No exclusion of data was made.
Replication	All experimental data was reliably reproduced in multiple independent experiments as indicated in the figure legends.
Randomization	Randomization is irrelevant to the present work since cell or animal samples were not compared across different conditions.
Blinding	For subcellular localization patterns of the long noncoding RNA (lncRNA) MALAT1 in HeLa cells experiments, data collection and analysis were performed by a person blinded to the treatment groups. Other experiments cannot be blinded due to objective factors, but this does not affect the conclusion of the article.

Reporting for specific materials, systems and methods

We require information from authors about some types of materials, experimental systems and methods used in many studies. Here, indicate whether each material, system or method listed is relevant to your study. If you are not sure if a list item applies to your research, read the appropriate section before selecting a response.

Materials & experimental systems

n/a	Involved in the study	
☐	☒	Antibodies
☐	☒	Eukaryotic cell lines
☒	☐	Palaeontology and archaeology
☐	☒	Animals and other organisms
☒	☐	Clinical data
☒	☐	Dual use research of concern

Methods

n/a	Involved in the study	
☒	☐	ChIP-seq
☒	☐	Flow cytometry
☒	☐	MRI-based neuroimaging

Antibodies

Antibodies used	Pol II antibody (Cat # ab5095, Polyclonal, 1:100, 1:1000, 1:5000, 1:10000 dilution) and NeuN antibody (Cat # ab177487, clone: EPR12763, 1:2000 dilution) were purchased from Abcam company. GABA antibody (Cat # PA5-32241, Polyclonal, 1:2000 dilution) was purchased from Invitrogen company. Flag antibody (Cat # 66008-3-Ig, clone: 2B3C4, 1:1000 dilution) was purchased from Proteintech company. CSFV E2 antibody (Cat # GTX60997, Polyclonal, 1:1000 dilution) and PCV2 Cap antibody (Cat # GTX128121, Polyclonal, 1:1000 dilution) were purchased from GeneTex company.
Validation	All antibodies were well-recognized clones in the field and validated by the manufacturers (see website). These antibodies are further validated and routinely used in our lab and in part antibody was also validated in previous publications work.<br><br>Pol II antibody (Cat # ab5095) https://www.abcam.com/rna-polymerase-ii-ctd-repeat-ysptsps-phospho-s2-antibody-ab5095.html.<br>(References: Gurumurthy A, Yu DT, Stees JR, Chamales P, Gavrilova E, Wassel P, et al. Super-enhancer mediated regulation of adult β-globin gene expression: the role of eRNA and Integrator. Nucleic acids research 2021, 49(3): 1383-1396.)<br><br>Flag antibody (Cat # 66008-3-Ig) https://www.ptgcn.com/products/Flag-tag-Antibody-66008-3-Ig.htm.<br>(References: Ling S, Yang S, Hu X, Yin D, Dai Y, Qian X, et al. Lentiviral delivery of co-packaged Cas9 mRNA and a Vegfa-targeting guide RNA prevents wet age-related macular degeneration in mice. Nature biomedical engineering 2021, 5(2): 144-156.)<br><br>NeuN antibody (Cat # ab177487) https://www.abcam.cn/neun-antibody-epr12763-neuronal-marker-ab177487.html.<br>(References: Wang YY, Zhao B, Wu MM, Zheng XL, Lin L, Yin DM. Overexpression of neuregulin 1 in GABAergic interneurons results in reversible cortical disinhibition. Nature communications 2021, 12(1): 278.)<br><br>GABA (Invitrogen,Cat # PA5-32241) https://www.thermofisher.cn/cn/zh/antibody/product/GABA-Antibody-Polyclonal/PA5-32241.<br>(References: Ryu B, Nagappan S, Santos-Valencia F, Lee P, Rodriguez E, Lackie M, et al. Chronic loss of inhibition in piriform cortex following brief, daily optogenetic stimulation. Cell reports 2021, 35(3): 109001.)<br><br>CSFV E2 antibody (Cat # GTX60997) https://www.genetex.cn/Product/Detail/CSFV-E2-protein-antibody/GTX60997.<br><br>PCV2 Cap antibody (Cat # GTX128121) https://www.genetex.cn/Product/Detail/Porcine-circovirus-type-2-PCV2-Capsid-antibody/GTX128121.<br>(References: Tsai GT, Lin YC, Lin WH, Lin JH, Chiou MT, Liu HF, et al. Phylogeographic and genetic characterization of porcine circovirus type 2 in Taiwan from 2001-2017. Scientific reports 2019, 9(1): 10782.)

Eukaryotic cell lines

Policy information about cell lines and Sex and Gender in Research

Cell line source(s)	PK-15 (ATCC, CCL-33), HeLa (ATCC CCL-2), HEK293T (ATCC, CRL-1573), BHK (ATCC, PTA-4506), A549 (ATCC, CCL-185), human foreskin fibroblast (HFF) (ATCC, SCRC-1041), LNCaP (ATCC, CRL-1740), PC3 (ATCC, CRL-1345), and THP-1 (ATCC, TIB-202) were obtained from the American Type Culture Collection (ATCC).
Authentication	The cell lines were routinely authenticated by growth curve analysis and morphology check using microscope.
Mycoplasma contamination	All cell lines were assessed regularly to ensure they were free of mycoplasma contamination.
Commonly misidentified lines (See ICLAC register)	No commonly misidentified cell lines were used.

Animals and other research organisms

Policy information about studies involving animals; ARRIVE guidelines recommended for reporting animal research, and Sex and Gender in Research

Laboratory animals	Wild-type CD-1 male mice (4 weeks old), wild-type C57BL/6 male mice (8 weeks old), and wild-type AB zebrafish (Danio rerio) embryos at 27 hours post-fertilization (hpf).
Wild animals	No wild animals were used.
Reporting on sex	We do not need to consider the sex of the animal in the study design because the method we report are generally applicable.
Field-collected samples	The study did not involve samples collected from the field.
Ethics oversight	This study was conducted according to the guidelines for experimental animals of the Research Ethics Committee of Huazhong Agricultural University. The use of animal samples was approved by the Scientific Ethic Committee of Huazhong Agricultural University (HZAUMO-2022-0063)

Note that full information on the approval of the study protocol must also be provided in the manuscript.
